# Syndrome de compression traumatique: à propos d'un cas

**DOI:** 10.11604/pamj.2014.19.242.3361

**Published:** 2014-11-04

**Authors:** Brahim Boukatta, Abderrahim El Bouazzaoui, Nawfal Houari, Hamid Jiber, Hicham Sbai, Nabil Kanjaa

**Affiliations:** 1 Université Sidi Mohammed Ben Abdellah, Service de Réanimation polyvalente du CHU Hassan II de Fès, Maroc; 2 Université Sidi Mohammed Ben Abdellah, Service de Chirurgie Vasculaire du CHU Hassan II de Fès, Maroc

**Keywords:** Crush syndrome, rhabdomyolyse traumatique, insuffisance rénale aigue, dialyse, aponévrotomie, Crush syndrome, traumatic rhabdomyolysis, acute renal failure, dialysis, fasciotomy

## Abstract

Le crush syndrome correspond à l'ensemble des manifestations systémiques secondaire à une destruction des fibres musculaires striées. Il survient le plus souvent dans le cadre d'accidents graves tels que les catastrophes, accidents de travail et accidents de la voie publique. Il est responsable d'une hypovolémie, état de choc, hyperkaliémie, hypocalcémie, acidose métabolique et d'insuffisance rénale aigue. Le succès du traitement dépend largement de la rapidité de la prise en charge. Les principaux objectifs thérapeutiques sont la correction de l'hypovolémie, traitement de l'hyperkaliémie, l'alcalinisation et la prévention de l'insuffisance rénale. L'utilisation de garrots compressifs doit être réservée au seul contrôle d'hémorragies importantes. Dans cet article, nous rapportons le cas d'un jeune patient de 20 ans ayant présenté un crush syndrome à la suite d'un accident de la voie publique. L’évolution était favorable, mais une amputation du membre écrasé était nécessaire.

## Introduction

Le syndrome de compression traumatique ou crush syndrome a été décrit pour la première fois par Bywaters et Beall au cours du bombardement de Londres lors de la deuxième guerre mondiale en 1941 [1]. Il correspond à un syndrome clinico-biologique secondaire à une rhabdomyolyse par destruction des fibres musculaires striées dont le contenu est libéré dans la circulation générale [2]. Il est responsable d'une morbi-mortalité importante, d'où l'intérêt d'une prise en charge rapide sur le lieu même de l'accident. Le crush syndrome survient le plus souvent dans le cadre d'accidents graves tels que les accidents de la voie publique, accidents de travail ou lors des catastrophes. Dans cet article, nous rapportons un cas de crush syndrome chez un jeune patient à la suite d'un accident de la voie publique.

## Patient et observation

Mr E.D âgé de 20 ans, sans antécédents pathologiques particuliers, a été victime le 25 Février 2013 d'un polytraumatisme suite à un accident de la voie publique. Motocycliste non casqué ayant été heurté par un camion, avec notion de perte de connaissance initiale et un écrasement du membre inférieur gauche pendant une durée non précisée. Le patient a été transféré par une ambulance non médicalisée aux urgences du CHU Hassan II de Fès. L'examen à son admission au service d'accueil des urgences, trouvait un patient agité, pâle, polypneique à 25 cycles/min, en état de choc hémorragique avec une pression artérielle à 70/40mmHg et une fréquence cardiaque à 145 batt/min. L'examen neurologique trouvait un patient agité, pupilles égales et réactives, sans signes de localisation. L'examen pleuro-pulmonaire était sans particularité. L'abdomen était souple et le bassin sans particularité. L'examen des membres trouvait une fracture ouverte du fémur gauche avec plaie saignante et abolition des pouls périphériques. Après monitorage, le patient a bénéficié d'une oxygénothérapie, prise de deux voies veineuses périphériques de gros calibre, d'un remplissage vasculaire par des cristalloïdes, d'une transfusion par trois culots globulaires O- et quatre plasmas frais congelés (PFC). Après stabilisation, un angioscanner des membres inférieurs a été réalisé, il a mis en évidence une section complète de l'artère poplitée gauche au niveau de sa partie sus-articulaire du genou. Le reste du bilan paraclinique a montré une tomodensitométrie cérébrale, une radiographie thoracique et une échographie abdominales normales. L'hémoglobine était à 7,2 g/dl après transfusion, les plaquettes à 206000 éléments/mm3, les globules blancs à 18000 éléments/mm3, une urée à 0,33g/l une créatinémie à 18 mg/l, un TP à 40%, des créatines phosphokinase (CPK) à 2075 UI/l et une troponine à 1,85ng/ml. Le patient a été admis au bloc opératoire.

Après induction en séquence rapide, le patient a bénéficié d'un monitorage standard, avec prise d'une voie veineuse centrale jugulaire interne droite. L'exploration chirurgicale a mis en évidence une section - contusion de l'artère poplitée avec des berges déchiquetées. Un pontage avec greffon veineux entre l'artère poplitée et le tronc tibio-péronier a été réalisé, puis le geste chirurgical a été complété par la mise en place d'un fixateur externe pour la fracture fémorale et des aponévrotomies de décharges. En peropératoire, le patient a présenté un saignement estimé à 1000ml, une instabilité hémodynamique ayant nécessité le recours aux fortes doses de drogues vasoactives, avec 1.5μg/kg/min de noradrénaline et 10 μg/kg/min de dobutamine. Le patient a bénéficié d'une transfusion par quatre culots globulaires. La durée de l'intervention était de trois heures. Le patient a été transféré ensuite au service de réanimation polyvalente. Le patient a été mis sous sédation avec midazolam et fentanyl, héparinothérapie curative, drogues vasoactives avec noradrénaline à 1,7μg/kg/min et dobutamine à 15μg/kg/min, remplissage vasculaire avec cristalloïdes et gélatines fluides modifiées et antibioprophylaxie par amoxicilline et acide clavulanique. Le postopératoire immédiat a été marqué par la survenue de plusieurs complications avec notamment, une insuffisance rénale aigue avec oligurie, des urines foncées, une urée à 0,58g/l et une créatinémie à 21mg/l, une acidose métabolique, avec un pH à 7,16, des HCO3- à 17,2 mmol/l, un excès de base à -10,5mmol/l et une PaCO2 à 50mmHg, une rhabdomyolyse avec des CPK à 4885 UI/l et une hyperkaliémie à 6 mmol/l, une anémie avec un taux d'hémoglobine à 5,4g/dl, une thrombopénie à 38000éléments/mm3 et une élévation de la troponine à 2,41 ng/ml. Le patient a bénéficié d'une transfusion par 5 culots globulaires et 5 PFC, d'un remplissage vasculaire par du sérum salé physiologique 0,9%, d'une alcalinisation par bicarbonates de sodium et d'une diurèse forcée par du mannitol. Douze heures après, le patient a présenté une ischémie du membre opéré, ce qui a nécessité une amputation transfémorale. L’évolution a été marquée ensuite par une amélioration progressive de l’état hémodynamique permettant un arrêt des drogues vasoactives le 3ème jour postopératoire, une reprise d'une diurèse normale avec éclaircissement des urines, une correction de l'acidose métabolique et une normalisation de la troponine, de la fonction rénale et du taux des plaquettes. Le patient a été extubé le 6ème jour puis il est sorti de l'hôpital 20 jours après.

## Discussion

La rhabdomyolyse correspond à une lyse de fibres musculaires striées, secondaire à une inadéquation entre les apports et les besoins métaboliques ou à une toxicité directe du myocyte par un toxique. Les rhabdomyolyses peuvent d’être d'origine ischémique, l'hypoxique, infectieuse, toxique ou post-traumatique. Ainsi, le syndrome d’écrasement ou crush syndrome survient essentiellement dans le cadre d'accidents graves, tels que les accidents de la voie publique, accidents de travail ou lors des catastrophes. Les mécanismes peuvent être intriqués, comme c'est le cas de notre patient, victime d'un écrasement, d'une hypovolémie et d'un état de choc hémorragique.

Sur le plan physiopathologique, l'ischémie entraine une diminution de la production de l'adénosine triphosphate (ATP) ce qui entraine une altération des mécanismes d'homéostasie cellulaire consommateurs d’énergie. Ainsi, une faillite des pompes Na-K ATPase-dépendantes entraine une accumulation de sodium et de l'eau dans le myocyte, responsable d'un oedème intracellulaire. Le dysfonctionnement de ces pompes entraine aussi une accumulation du calcium ionisé dans le cytosol des mitochondries, responsable de contractures et de crampes. L'altération de la perméabilité membranaire secondaire à la surproduction des radicaux libres, entraine un afflux massif du sel et de l'eau dans la cellule. Les muscles étant enveloppés entièrement par des aponévroses inextensibles, le développement de l'oedème s'accompagne d'une augmentation marquée de la pression interstitielle locale, comprimant les vaisseaux et les nerfs. En plus, l'extravasation entraine une hypovolémie. Le reperfusion après une période d'ischémie aggrave les lésions tissulaires. Le rétablissement d'un débit sanguin favorise le passage du contenu cellulaire dans la circulation générale, la production massive de radicaux libres par l'arrivée de l'oxygène au niveau du muscle strié ischémique et le déclenchement d'une réaction inflammatoire systémique. Tous ces facteurs aboutissent à un dysfonctionnement des cellules endothéliales, à une augmentation de la perméabilité aggravant les oedèmes et à des perturbations de la microcirculation. Au total, l'hypovolémie, la libération du contenu cellulaire (potassium, phosphore, myoglobine, créatine phosphokinase, acide urique) dans la circulation sanguine, la production de radicaux libres et de cytokines, la coagulation intravasculaire disséminée (CIVD) qui résulte de la libération du facteur tissulaire, favorisent l'extension de l'atteinte initiale vers d'autres organes. De ce fait, des atteintes cardiaques, hépatiques, pulmonaires (SDRA) ont été décrites. Tous ces phénomènes peuvent donc favoriser l'apparition d'un syndrome de défaillance multiviscérale. Les principaux mécanismes de l'insuffisance rénale aigue sont l'hypoperfusion rénale liée à l'hypovolémie, l'hypoxie de la médullaire responsable de la nécrose tubulaire aigue, l'obstruction des tubules rénaux par des cristaux de myoglobine et d'acide urique, dont la précipitation est favorisée par l'acidité des urines et la toxicité directe des radicaux libres sur les cellules tubulaires. L'instabilité hémodynamique liée au crush syndrome est multifactorielle, elle peut être expliquée par le saignement qui rentre dans le cadre du polytraumatisme, la séquestration liquidienne au niveau des masses musculaires lésées et qui peut atteindre jusqu’à 10 litres durant les 48 premières heures et la dépression myocardique secondaire à la libération des toxines, aux troubles ioniques (hyperkaliémie, hypocalcémie) et à l'acidose métabolique. La survenue du syndrome de détresse respiratoire aigue (SDRA) peut être expliquée par le traumatisme, la réaction inflammatoire systémique, l'embolie graisseuse et le remplissage vasculaire massif [3, 4].

Sur le plan clinique, on trouve essentiellement, des lésions cutanées avec des dermabrasions, des ecchymoses et des décollements cutanés. Le patient peut présenter des fractures fermées ou ouvertes. Les lésions nerveuses sont secondaires soit à l'ischémie, aux étirements ou à la section; elles intéressent surtout le nerf radial et le nerf sciatique. Les gros troncs vasculaires sont fréquemment atteints lors d’écrasement de membres, souvent en regard d’étirements ou de fractures comminutives, en particulier au niveau huméral proximal, au niveau du coude ou du genou. Les signes cliniques liés à l'atteinte musculaire et à la rhabdomyolyse (fatigue, douleurs) sont généralement masqués par les troubles de conscience, les urines deviennent foncées par la présence de la myoglobine. L'oedème musculaire est responsable du syndrome de loge avec des signes d'ischémie et des troubles sensitivo- moteurs. Le crush syndrome s'accompagne de manifestations générales, avec des signes d'hypovolémie, une oligo-anurie, un état de choc, des troubles neuropsychiques, une hyperventilation en rapport avec l'acidose métabolique et des arythmies liées à l'hyperkaliémie. Une coagulopathie de consommation et le SDRA peuvent également survenir en raison de la libération des radicaux libres lors de la reperfusion. Les autres lésions crâniennes, thoraciques, abdominales, pelviennes et osseuses sont en rapport avec le polytraumatisme. Notre patient a présenté des lésions cutanées, vasculaires, musculaires, osseuses associés à un état de choc hémorragique.

Sur le plan biologique, on trouve une hyperkaliémie, elle est secondaire à la libération du potassium intracellulaire, elle est précoce et engage le pronostic vital par les troubles de rythme cardiaque qu'ils entrainent. L'acidose métabolique est liée à l’état de choc et à l'insuffisance rénale, elle doit être corrigée activement car elle aggrave l'hyperkaliémie et favorise la précipitation intratubulaire de myoglobine en particulier. Les autres troubles métaboliques sont représentés par l'hypocalcémie, l'hyperphosphorémie, l'hypoalbuminémie, la myoglobinémie et la myoglobinurie. L’élévation de la créatinine phosphokinase (CPK) signe la nécrose musculaire, le pic de concentration n'apparaît qu'au 3ème jour après le traumatisme aigu. On peut parler de rhabdomyolyse si les CPK sont supérieures à 1000 UI/l. Des CPK à 7000 UI /l correspondent à une rhabdomyolyse modérée. Le risque d'insuffisance rénale est réel pour des CPK > 16000 UI/ l. Les troubles de l'hémostase cliniques et biologiques dans le cadre da la CIVD sont fréquents dans les syndromes d’écrasement, les principaux mécanismes responsables sont la spoliation et la consommation des facteurs de la coagulation dans le cadre du traumatisme et les incisions aponévrotiques de décharge, la dilution secondaire au remplissage, le syndrome inflammatoire réactionnel systémique, l'acidose métabolique, l'hypocalcémie et l'hypothermie [5].

L'incidence de l'insuffisance rénale dans les syndromes d’écrasement varie entre 31 et 52%. Le patient est le plus souvent oligo-anurique. Les principaux facteurs de risque de l'insuffisance rénale sont le délai de prise en charge, l'acidose métabolique sévère (bicarbonates < 17 mmol/l), un taux d'hémoglobine bas, une myoglobinurie et des CPK supérieures à 8500 U/l. Notre patient a présenté une acidose métabolique, une hyperkaliémie, une élévation des CPK et une insuffisance rénale.

Le traitement de la rhabdomyolyse traumatique doit être débuté sur le lieu même de l'accident et avant même la désincarcération de la victime. Le patient doit être transporté ensuite par une ambulance médicalisée vers une structure hospitalière adaptée, comportant une unité de soins intensifs avec possibilité d’épuration extrarénale. Les principaux objectifs thérapeutiques sont la correction de l'hypovolémie et de l'hyperkaliémie, la prévention de l'insuffisance rénale et de la coagulopathie et la stabilisation des lésions en rapport avec le polytraumatisme. La prise en charge impose une surveillance étroite de la fréquence cardiaque et respiratoire, de la pression artérielle invasive, du rythme cardiaque, de la saturation en oxygène, de la pression veineuse centrale, de la température, de la glycémie capillaire, de la pression de loges musculaires, du pH urinaire qui doit être supérieur à 6,5 et de la diurèse horaire, avec comme objectif un débit urinaire horaire de 200 à 300ml (3ml/kg/h) [6]. Le patient doit bénéficier de deux abords veineux périphériques de gros calibres, d'un remplissage vasculaire massif, qui doit être initié avant même la désincarcération de la victime. Le sérum salé physiologique 0,9% réchauffé constitue le soluté de remplissage de choix. Il est recommandé de commencer par un débit de 10 à 15ml/kg/h puis par 500ml/h pendant les premières 24 heures. Ainsi, le volume moyen de perfusion peut atteindre 10 à 20 litres par jour [7]. Les colloïdes, type hydroxyéthylamidon (HEA), trouvent leur place surtout en cas d’état de choc. Le Ringer lactate est contre-indiqué car il contient du potassium. L'hyperkaliémie constitue une urgence thérapeutique, elle nécessite une alcalinisation, un traitement diurétique le plus souvent par du mannitol 20% à la dose de 1 à 2 g/ kg sur les quatre premières heures et en cas de signes électriques, une injection de chlorure de calcium. La succinylcholine doit être évité en cas d'anesthésie générale.

En cas d'insuffisance rénale avec hyperkaliémie, acidose sévère et surcharge volémique une épuration extrarénale est nécessaire. Les diurétiques de l'anse sont à éviter car ils entrainent une acidification des urines. Le mannitol, en plus de son effet diurétique, permet une augmentation du volume vasculaire, une amélioration du débit cardiaque et urinaire; et une diminution de la pression au niveau des loges musculaires. Devant le risque néphrotoxique et de l'hyperosmolarité, la dose ne doit pas dépasser 200g/j et il doit être évité chez les patients anuriques [8]. L'alcalinisation par bicarbonates de sodium doit assurée un pH urinaire supérieur à 6,5 pour prévenir la précipitation de la myoglobine au niveau des tubules urinaires. Cette perfusion alcaline doit être maintenue pendant 3 jours en moyenne, temps nécessaire à l’élimination de la myoglobine. Le reste du traitement comprend, une prévention de l'hypothermie, une analgésie efficace, le plus souvent multimodale et une oxygénothérapie au masque pour lutter contre l'hypoxie. La ventilation mécanique peut être nécessaire en cas d’état de choc, d'hypoxie sévère ou de lésions cérébrales ou thoraciques en rapport avec le polytraumatisme. Une hypotension ne répondant pas au remplissage impose le recours précoce aux drogues vasoactives. En cas de suspicion de lésions du rachis cervical, il faut mettre une minerve. Les fractures de membres nécessitent une immobilisation et les plaies hémorragiques un pansement compressif. Le garrot compressif est dangereux, car il aggrave l'ischémie musculaire, il doit être réservé aux hémorragies artérielles importantes. L'antibiothérapie et la prophylaxie antitétanique doivent répondre aux recommandations en matière des traumatismes ouverts. La transfusion sanguine est souvent nécessaire, elle vise une hémoglobine entre 9 et 10g/dl. Le traitement systématique de l'hypocalcémie est inutile, en dehors des hypocalcémies liées au trouble d'hémostase.

Certaines études ont montré l'intérêt de l'oxygénothérapie hyperbare dans l'amélioration de l'hypoxie liée au crush syndrome [9, 10]. La prise en charge chirurgicale du membre écrasé se fait généralement en 3 temps: stabilisation de la fracture, le plus souvent par un fixateur externe en raison des lésions cutanées et musculaires; réparation des lésions vasculaires, le plus souvent par la réalisation des pontages et enfin les aponévrotomies de décharges pour lutter contre le syndrome de loges, celui-ci est définie par une pression de loges supérieure à 30mmHg avec des signes de souffrance vasculo-nerveuses. L'amputation du membre reste une décision difficile, qui nécessite une discussion multidisciplinaire, incluant le chirurgien, l'anesthésiste, la famille et si possible, le patient. Une fois la décision prise, l'opérateur doit respecter les bonnes règles en choisissant le niveau d'amputation pour permettre au patient de tirer le meilleur bénéfice possible d'un appareillage performant. Notre patient malheureusement a bénéficié d'une amputation après échec du traitement conservateur.

## Conclusion

L’écrasement traumatique de membre est d'abord une pathologie régionale grave qui peut rapidement devenir systémique et mettre en jeu le pronostic vital par le mécanisme d'ischémie/reperfusion du membre écrasé. Les principales causes de décès sont le choc hypovolémique, l'hyperkaliémie, l'insuffisance rénale, la toxicité cardiaque aigue et le syndrome de défaillance multiviscérale. La prise en charge doit être rapide et agressive sur le lieu même de l'accident, elle doit être focalisée sur le traitement et la prévention de l'hypovolémie, de l'hyperkaliémie et de l'insuffisance rénale.
